# An Extenics-TRIZ integrated RFPS model for different object of design requirements

**DOI:** 10.1371/journal.pone.0316138

**Published:** 2025-01-02

**Authors:** Fangzhi Gui, Jing Zhou, Xiangdong Sun, Jianfei Lu, Sheng Cao

**Affiliations:** 1 School of Mechanical Engineering, Nanchang Institute of Technology, Nanchang, Jiangxi, China; 2 School of Electrical Engineering, Xian University of Technology, Shaanxi, Xian, China; 3 Zhejiang Chendiao Machinery Co., Ltd, Lishui, Zhejiang, China; 4 Faculty of Engineering, Technology and Built Environment, UCSI University, Kuala Lumpur, Malaysia; Cyprus International University Faculty of Engineering: Uluslararasi Kibris Universitesi Muhendislik Fakultesi, TÜRKIYE

## Abstract

Extending product life is one of the effective ways to reduce the waste of resources. However, many unsatisfactory products are scrapped because of a lack of adequate performance. The product should be improved and upgraded innovatively, and the existing upgradable products may create more economic benefits for the longer product life cycles. This paper proposed a product innovative design and product upgrade employing an Extenics-TRIZ Integrated requirement-function-principle-structure (RFPS) model, which aims at complex requirement flexibility with easy-to-use design process when the product needs a redesign. Here, the requirement flexibility refers to the ability of a design object to adapt its design levels. There are two design strategies: the extension analysis methods are utilized to map the top-level requirements to functions, principles, and structures requirements, and then the TRIZ is used to handle the design problems according to the objects on different levels. This design knowledge is summarized as RFPS, and it can be reused in computer-aided innovation further. A case study for a cutting table is illustrated to the innovation and upgrade, and it indicates the effectiveness for designers to implement the design methodology.

## Introduction

Improving product lifetime is a meaningful approach to reduce environmental issues [1]. However, some products are cast aside because they won’t meet customers’ needs any longer, and it will result in consuming more natural resources and generating more wastes [2]. Normally, upgrading these unsatisfactory products is a simple, efficient and economical way to meet customer needs again. The diversity of innovation ideas and thoughts was observed, and it was generally a brainstorming process, which is simple but always inefficient and resource-consuming due to the uncertainty in the quantity and quality of ideas [3]. Therefore, it is of great significance to formulate a set of effective and convenient innovation design for product innovation and upgrades, and it is also one of the issues to accelerate manufacturing industrial upgrading. Meanwhile, modern knowledge-driven and data-drive techniques are transforming many disciplines by knowledge acquisition, uncovering patterns in data, etc., and supporting human decision-making.

To make the design intelligent, design researchers and experts have established relevant knowledge representation schema to describe the product design development. Suh proposed a conceptual model for the four design steps of a "thinking design machine" by using the Axiomatic Design (AD) approach [4, 5]. The four steps are respectively corresponded to Customers Attributes (CAs), Functional Requirements (FRs), Design Parameters (DPs) and Process Variables (PVs), which are defined as Customer Domain, Function Domain, Physical Domain and Process Domain, so that the design roadmap is the mutual mapping process between two adjacent design domains. Quality Function Deployment (QFD) is a customer-driven product development roadmap. QFD is a helpful tool to translate the voice of customers (VoC) into technical languages [6], and the technical languages gradually map to engineering characteristics (EC), parts characteristics (PC), process operations and production requirements with the House of Quality (HoQ), forming a complete product development roadmap.

AD and QFD proposed the concept of a step-by-step implementation and the process object sets, but did not form the knowledge representation scheme. Therefore, a large number of other scholars have carried out more design analysis processes on the basis of knowledge representation. Gero [7] proposes a knowledge-oriented Function-Behavior-Structure (FBS) representation model. The behavior (B) is a bridge between function (F) and structure (S), that is, it is the principle of function realization, and it is also reflected by the structure of the product. Therefore, behaviors are divided into expected behavior (Be) and the behavior derived from structures (Bs). Based on FBS, some scholars have conducted further research on it. Helfman [8] provided a structure–function pattern for biomimetic applications, and the TRIZ method was used for modeling biological systems of the biomimetic design process. Umeda [2, 9] proposed function-behavior-state model, in which the “state” includes structures and other physical knowledge about structures. Some other representation models, such as FPS [10], SBF [11], RFBSE [12] etc., are put forward for a similar intent. In recent years, the requirements from users, designers or policy have been placed greater emphasis on product design, there some research is incorporating these requirements into FBS. For example, Christophe [13] proposed an extended RFBS, and the abbreviated letter R refers to requirements, which means taking customer needs or requirements into account. Fu et al [14] proposed a constraint-driven function-behavior-structure design process, which converted customer requirements into design constraints. Luo et al [15] proposes an interval-valued Pythagorean fuzzy set-based FBS model integrating AHP and HOQ methods to reduce the influence of user requirement ambiguity. Li and Lou et. al [16] proposed a cerebellar operant conditioning-inspired constraint satisfaction approach to solve the mapping process from behaviors to structures to facilitate the cognitive activities of designers. Li and Tang et al [17] presented a framework of the dynamic function-behaviour-structure cell model, which generated the optimal scheme for open design based on co-designer involvement. Zheng et al [18] presented a function-structure synthesis approach based on case-based reasoning to meet the requirements of the low carbon policy. Although Zhu et al [19] proposed the RFPS model, there was no innovative design based on classification according to requirements. These studies consider the requirements from various parties, but they do not classify the requirements according to innovation directions to improve the efficiency. At the same time, it can be seen from the above points that: (1) Requirements, Functions, Principles, Behaviors, and Structures are considered as key elements for design. The CAs, FRs, and DPs in AD and the VoC and EC in HoQ can also be transformed to one of them. (2) There is a certain mapping relationship between these key elements.

This paper aims to propose a design methodology for product upgrade and innovation. In QFD, the requirements are mapped into VoC, EC and PC, etc., and they represent the most key objects for product design in different stages. It reminds us that the object of innovation or upgrade is one of the important considerations for product upgrade or innovation. It can be also seen that functions and structures are key elements in product design as many researchers believed, and the mapping from functions to structures can be straightforward or indirect. Motivated by the above facts, product upgrade and innovation processes are proposed with RFPS design knowledge representation scheme, which divides the innovative design requirements R into different levels of problems according to the upgrading objects, refering to function F, principle P, or structure S. For these problems, TRIZ is an effective theory of inventive problem solving with a lot of toolkits that summarize the successful experiences of past solutions, such as the inventive principle, Su-Field model, Effects, and so on. In general, TRIZ can independently solve general conflicting issues or combine with other methods to improve the effectiveness of solutions [20–22].

In the remainder of this paper, the requirements are classified into the requirements for function, principle and structure innovation, and the Extension analysis methods are used to map top-level requirements to function, principle and structure requirements in Section 2. In section 3, the RFPS model is set forth, and different design procedures are shown to solve different design object with TRIZ; and in section 4, a case study of cutting table is presented to show the methodology. In the last sections 5 and 6, some conclusions about this methodology will be drawn, along with some discussion on future work.

## RFPS knowledge representation and mapping

### Design objects from requirements to functions, principles and structures

Requirements, such as the low cost or low carbon emission, are the motive force behind product innovation. There are two main requirements analysis methods for product design at present—QFD-based [23] and Kano-based [24] method. Kano method focuses on user’s perception while QFD method pays close attention to product performance, and both of them aim to increase users’ satisfaction, so that they are a kind of “adaptive requirements”. On the other hand, from the perspective of the designers, product should also actively improve with the new technology, that is, the product needs to be updated by “creating requirements”. For “adaptive requirements”, there are two innovative ways—One is to optimize the structures under certain constraints continuously, such as the further improvement of surface accuracy, etc.; the other is to change the principle with the same function realization, such as the screen display from the CRT to LCD technology, etc. For “creating requirements”, it is mainly through changing the old functions or adding new functions of product. The main way is through the transfer of technical fields, such as applying military GPS technology to civil navigation.

**Fig 1:**
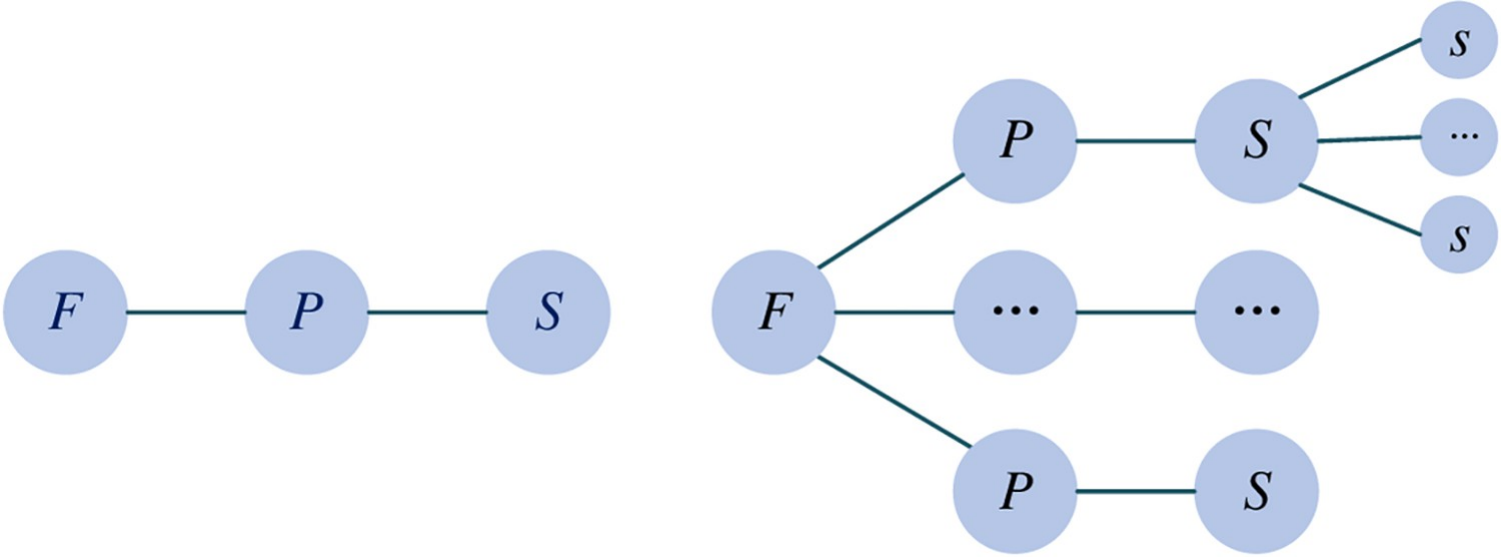
Mapping relation of *F*, *P*, and *S*.

The function of a product represents an abstract delineation of its operational inputs and outputs, encompassing both the overarching function and the essential sub-functions that facilitate the top-function. The structure, conversely, represents the tangible manifestation of the product design’s foundational object—a structure may manifest as a part, a component, or a module, serving as the vehicle for function and the embodiment of the underlying principles. Thus, the principle serves as a pivotal bridge, linking the domain of function to that of structure, encompassing the spectrum from physical phenomena and scientific methodologies. In the context of functional design, a single function may be associated with one or multiple principles. For instance, the function of "reducing noise" can be addressed through noise reduction at the transmission stage, at the source, or at the receiving end. Conversely, a single principle may correspond to one or several structures. This mapping relationship is depicted in Fig 1. Given this interplay, the function, principle, and structure are identified as the three fundamental entities for product enhancement and innovation, tailored to the specific design requirements. This tripartite approach ensures a comprehensive understanding of the product and provides a structured pathway for innovation and improvement.

**Fig 2:**
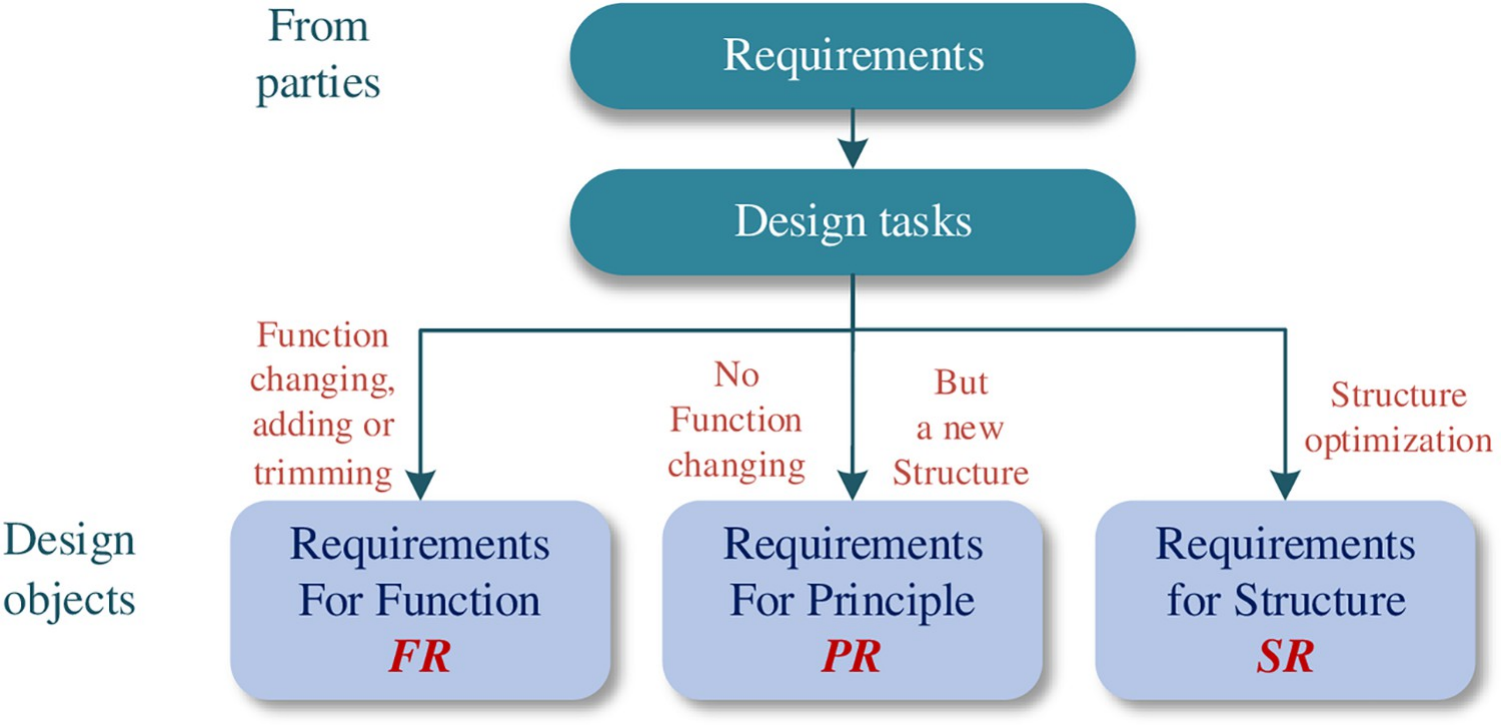
Design objects with requirements classification.

Design tasks are divided into different types of designs according to the degree of innovation, and the majority of tasks are adaptations and variations on existing designs. Based on this, combined with the above hierarchical FPS mapping relation, all the design requirements goals are classified into the three categories first to foster and guide the abilities of designers: (1) Requirements for Function Innovation (*FR*), (2) Requirements for Principle Innovation (*PR*), and (3) Requirements for Structure Innovation (*SR*), shown as Fig 2.

### Extension analysis methods

Before extension analysis, the basic element [25] should be introduced first. The basic element is used for formalized description of matters, affairs and relations, and expressed as

$$B=(\begin{array}{ccc} O, & c, & v \end{array})=[\begin{array}{ccc} O, & c_{1}, & v_{1}\\ & \vdots & \vdots \\ & c_{m}, & v_{m} \end{array}]$$ (1)

Where *O* is the object, and *c* is a characteristic of *O*, and *v* is a measure of *O* about *c*. The simplified basic element *B* = (*O*, ⋯) can be used to represent the unknown or indescribable characteristics. These data about requirements, functions, principles and structures are expressed as basic element and stored in database.

For a complex design requirement, some analysis methods are needed to obtain the simple design objects. Extension analysis methods, including divergent analysis, correlative analysis, implicative analysis, and opening-up analysis, are specific tools to analyze these objects [26, 27].

#### Divergent analysis

The divergent analysis is the generalization and expansion of objects. It is mainly aimed at obtaining other similar objects from the divergence of "one object with multiple characteristics" and "same characteristic of multiple objects." Divergent analysis includes analysis based on objects, characteristics, and values. They are defined as follows:

- Object-based divergence: The mapping of basic element *B*_0_ to a set of basic elements *B* = {*B*_1_, *B*_2_, …, *B*_*n*_}(*n*≥2) is an object-based divergence, if and only if the objects of these basic elements are the same;
- Characteristic-based divergence: The mapping of basic element *B*_0_ to a set of basic elements *B* = {*B*_1_, *B*_2_, …, *B*_*n*_}(*n*≥2) is a Characteristic-based divergence, if and only if the characteristics of these basic elements are the same; and
- Value-based divergence: The mapping of basic element *B*_0_ to a set of basic elements *B* = {*B*_1_, *B*_2_, …, *B*_*n*_}(*n*≥2) is a value-based divergence, if and only if the values of these basic elements are the same.

These divergences can be formulated as:

$$B=(\begin{array}{ccc} O, & c, & v \end{array})┤\{\begin{array}{l} \left\{(\begin{array}{ccc} O_{i}, & c, & v_{i} \end{array}),i=1,2,\cdots ,n\}=\{\left(\begin{array}{ccc} O_{1}, & c, & v_{1} \end{array}),(\begin{array}{ccc} O_{2}, & c, & v_{2} \end{array}),\cdots ,(\begin{array}{ccc} O_{n}, & c, & v_{n} \end{array}\right)\right\}\\ \left\{(\begin{array}{ccc} O, & c_{i}, & v_{i} \end{array}),i=1,2,\cdots ,n\}=\{\left(\begin{array}{ccc} O, & c_{1}, & v_{1} \end{array}),(\begin{array}{ccc} O, & c_{2}, & v_{2} \end{array}),\cdots ,(\begin{array}{ccc} O, & c_{n}, & v_{n} \end{array}\right)\right\}\\ \left\{(\begin{array}{ccc} O, & c, & v_{i} \end{array}),i=1,2,\cdots ,n\}=\{\left(\begin{array}{ccc} O, & c, & v_{1} \end{array}),(\begin{array}{ccc} O, & c, & v_{2} \end{array}),\cdots ,(\begin{array}{ccc} O, & c, & v_{n} \end{array}\right)\right\} \end{array}$$ (2)

Where the symbol “┤” represents divergence.

#### Correlative analysis

The correlative analysis is a method to analyze the relation for better and clearer comprehension among all the basic elements, which reflects the mechanism of correlation and interaction. If there is a certain relation between two or more basic elements, these basic elements can be defined as correlation, and the general definition is formulated as

$$B_{1}∼B_{2}$$ (3)

Where the symbol “~” represents the correlation.

**Fig 3:**
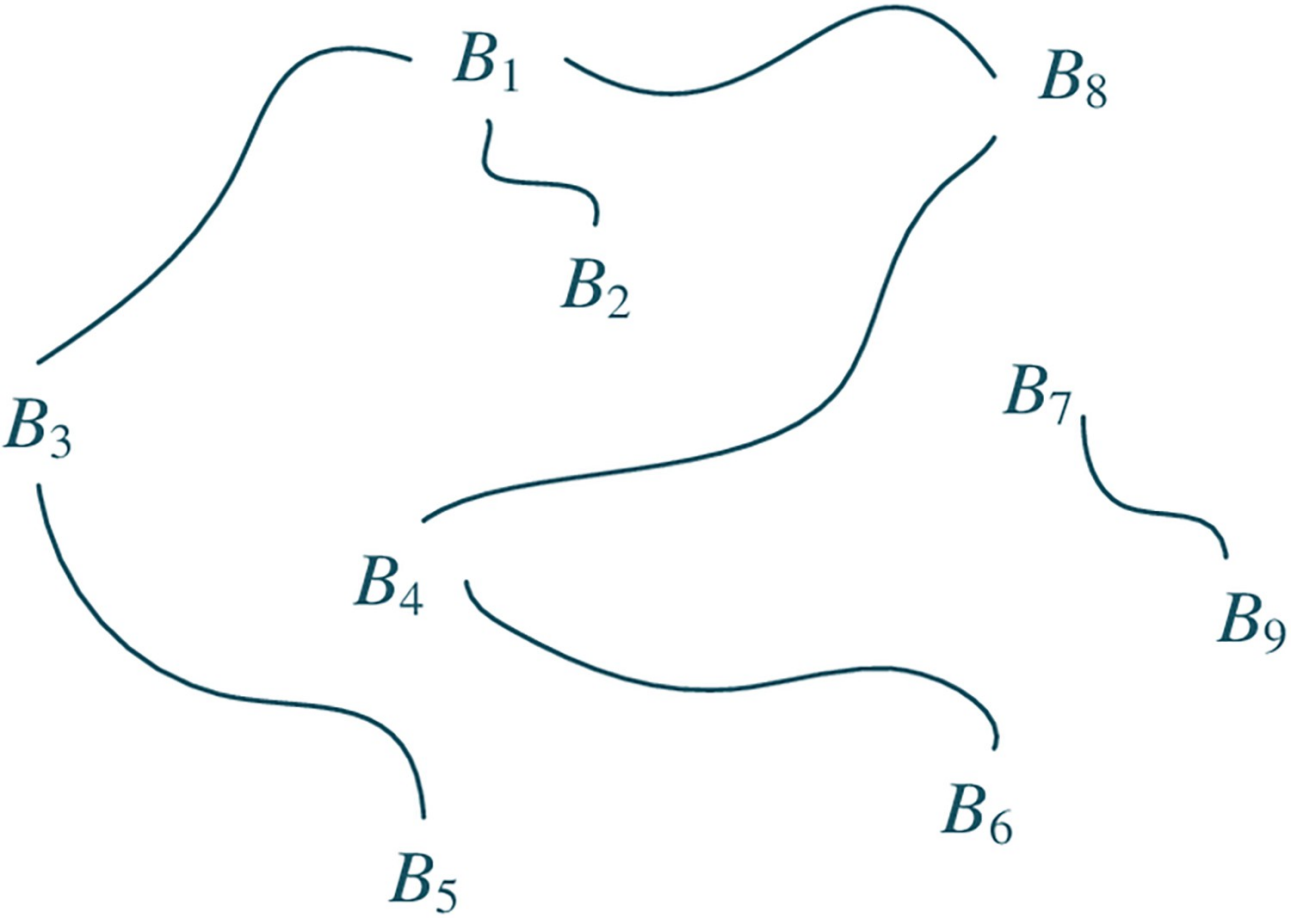
Correlation network.

The correlation among some basic elements can be represented in a network as Fig 3.

#### Implicative analysis

The implication analysis is based on the connection of basic elements, which refers to a hierarchical relation, so there is a pair of superior and inferior. If the inferior is the premise of the superior, that is, the implementation of superior basic element must have the implementation of inferior basic element, then the inferior implies the superior, and it is expressed as

$$B_{1}\Rightarrow B_{2}(\mathrm{or}\;B_{1}@\Rightarrow B_{2})\;\mathrm{or}\;B_{2}⇐B_{1}(\mathrm{or}\;B_{2}@⇐B_{1})$$ (4)

Where *B*_1_ refers to the inferior, and *B*_1_ refers to the superior. The symbols “⇒” and “⇐” represent the implication, and the symbol “@” represents the implementation of basic element.

Specifically, there are two types of implication—AND implication and OR implication.

**Fig 4:**
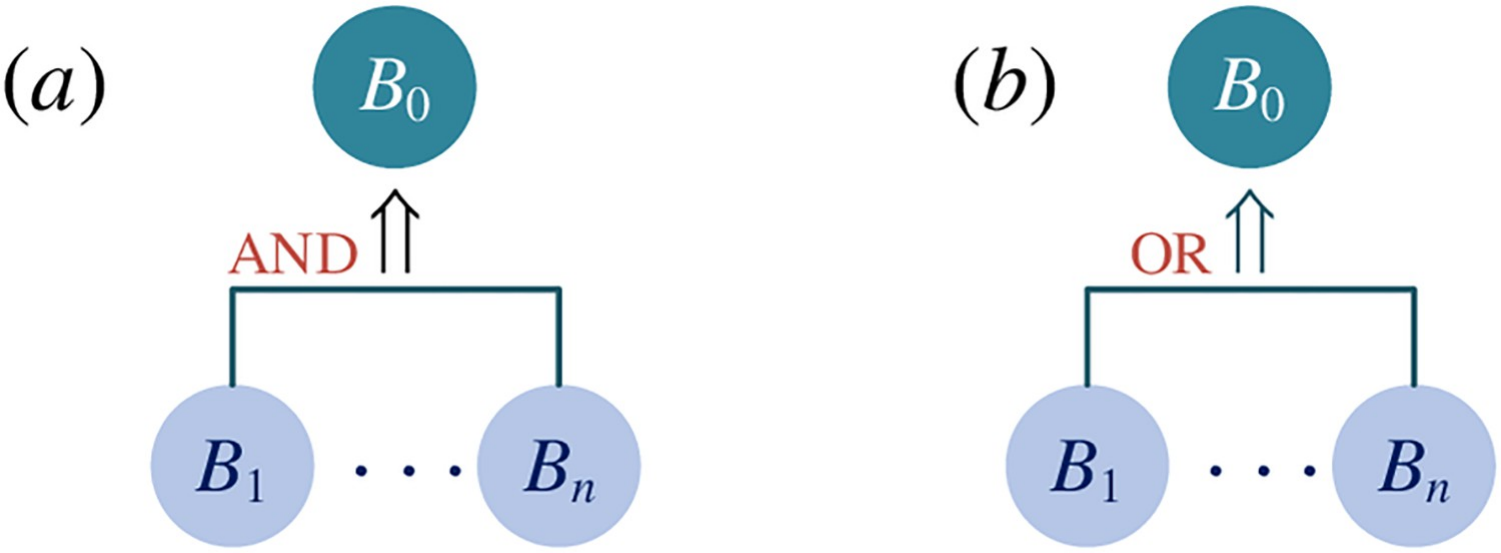
Implicative analysis diagram (a) AND implication (b) OR implication.

- *AND implication*. It is an AND implication that a set of basic elements *B* = {*B*_1_, *B*_2_, …, *B*_*n*_}(*n*≥2) implies the basic element *B*_0_, if and only if the basic element *B*_0_ is implemented with the all basic elements in the set *B* implemented, as shown in Fig 4(Aa), and it is expressed as (*B*_1_∧*B*_2_∧…∧*B*_*n*_)⇒*B*_0_ and
- *OR implication*. It is an OR implication that a set of basic elements *B* = {*B*_1_, *B*_2_, …, *B*_*n*_}(*n*≥2) implies the basic element *B*_0_, if and only if the basic element *B*_0_ is implemented with the any basic element in the set *B* implemented, as shown in Fig 4(B), and it is expressed as (*B*_1_∨*B*_2_∨⋯∨*B*_*n*_)⇒*B*_0_

#### Opening-up analysis

The opening-up analysis is a method that takes the possibility of composability, decomposability, scalability into account. Therefore, there are 3 types of opening-up analysis, such as composable or decomposable analysis and scalable analysis.

*a) Composable analysis*. For any basic element *B*_*i*_, there is at least one other basic element *B*_*j*_ that can be composed into B with *B*_*i*_ according to the objects or characteristics. That is

$$B=B_{i}⊕B_{j}=\{\begin{array}{l} (\begin{array}{ccc} O_{i}, & c_{i}⊕c_{j}, & v_{i}⊕v_{j} \end{array}),if\;O_{i}=O_{j},c_{i}\neq c_{j}\\ (\begin{array}{ccc} O_{i}⊕O_{j}, & c_{i}, & v_{i}⊕v_{j} \end{array}),if\;O_{i}\neq O_{j},c_{i}=c_{j}\\ \left[\begin{array}{rrr} O_{i}⊕O_{j}, & c_{i}, & v_{i}⊕v_{j}\\ & c_{j}, & v_{i}⊕v_{j} \end{array}\right],if\;O_{i}\neq O_{j},c_{i}\neq c_{j} \end{array}$$ (5)

Where the symbol “⨁” represents the composition;

*b) Decomposable analysis*. For any basic element *B*, it can be decomposed into a set of basic elements *B* = {*B*_1_, *B*_2_, …, *B*_*n*_}(*n*≥2) under certain condition. That is

$$B//\{B_{1},B_{2},\ldots ,B_{n}\}$$ (6)

Where the symbols “//” represents the decomposition; and

*c) Scalable analysis*. For any basic element *B* = (*O*, *c*, *v*), it can be scaled into another basic element *αB* at multiple of *α* (*α* > 0) for change of the value *v*. That is

αB=(αO,c,αv) (7)

Where *αB* and *αO* are the basic element and object after scaled respectively.

### Requirements mapping

According to the description of knowledge representation of abovementioned requirements, functions, principles and structures, and the extension analysis methods, a hierarchical analysis can be performed for specific objects with these methods.

The mapping process consists of two objects and a mapping method. Mapping from *A* to *B*, for example, can be formulated as *A*→*B*. The mapping objects and method for *R*, *FR*, *PR* and *SR* are shown in Table 1 where the first vertical is the former item *A*, the first horizontal is the later item *B*, and the other data in the table are available mapping methods.

**Table 1:** Mapping objects and available methods.

object	*R*	*FR*	*PR*	*SR*
*R*	~, ┤, ⇐, ⨁, //, *α*	┤, ⇐	┤, ⇐	┤, ⇐
*FR*	/	~, ┤, ⇐, ⨁, //, *α*	┤, ⇐	/
*PR*	/	/	~, ┤, ⇐, ⨁, //, *α*	┤, ⇐
*SR*	/	/	/	~, ┤, ⇐, ⨁, //, *α*

According to the Table 1, It should be noted that the requirements *R* can be mapped not only to functions *FR* directly, but also to principles *PR* and structures *SR* with different extension methods. For requirements for function or principle innovation, it is also achieved through structure changing and optimizing ultimately.

## RFPS method

### Workflow

**Fig 5:**
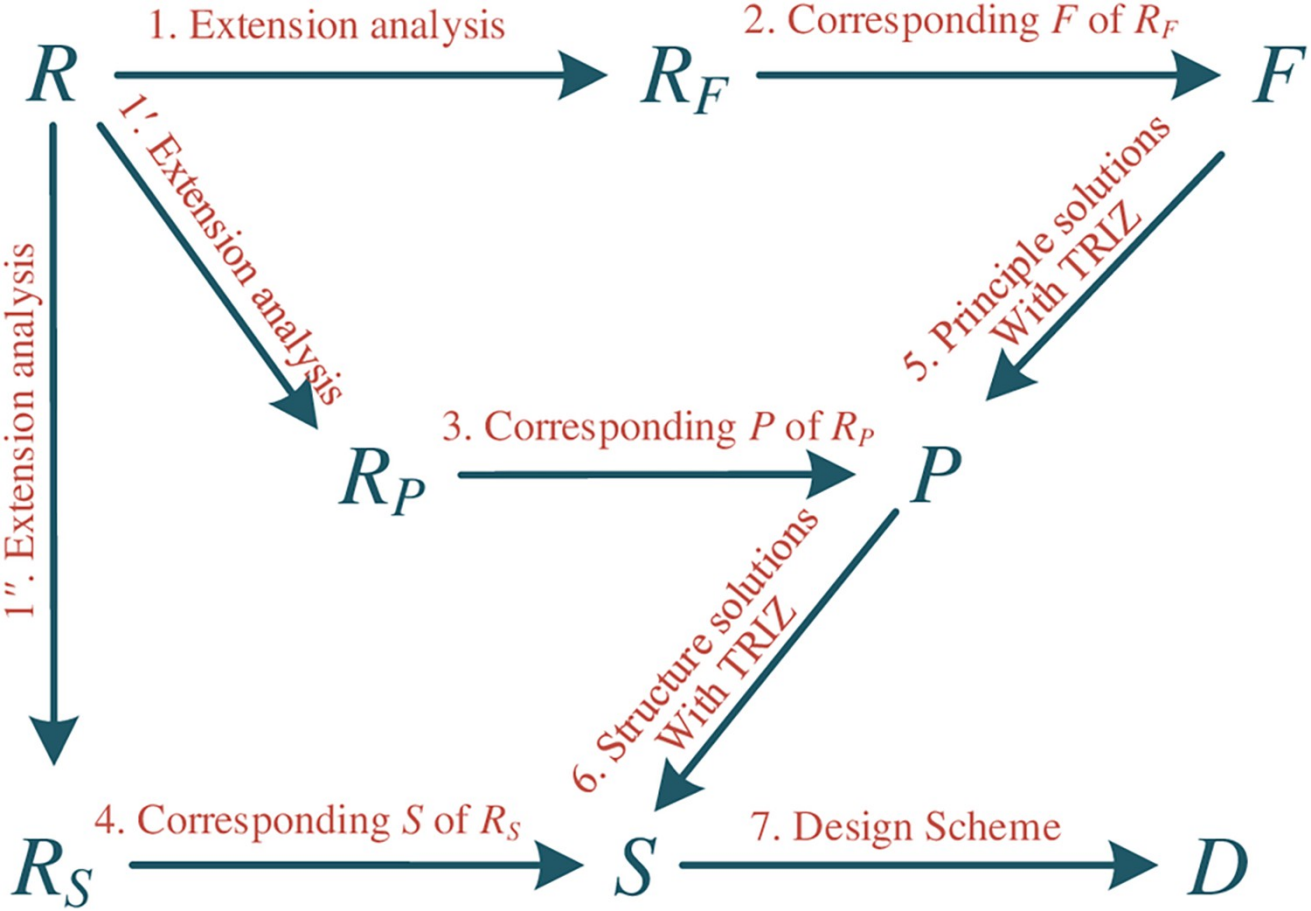
RFPS workflow.

Based on the content in Section above, the design methodology is proposed here (see Fig 5), which is referred as RFPS, and the detail processes are explained in Table 2. It can be seen that:

a) The design model starts with requirement *R*, which represents the innovative intention;

b) The process 1 in Fig 5 means that innovative design intentions are classified into innovative requirements for structures, principles and functions according to the extension analysis result.

c) The process 2 in Fig 5 means that the requirements for function innovation (*FR*) are mapped into functions (*F*). The functional design problem is modeled and there obtains an innovative product design scheme with TRIZ, and then to choose an innovative principle solutions and design a novel structure solution. This process can be briefly described as “function-principle-structure (FPS)”, and it is shown in Fig 5 as steps 1→2→5→6→7 in turn. The requirements for function innovation are detailed in following part.

d) The process 3 in Fig 5 means that the requirements for principle innovation (*PR*) are mapped into principles (*P*). The principle design problem is modeled and then there obtains an innovative product design scheme by searching the effects database and then to design a novel structure solution. This process can be briefly described as “principle-structure (PS)” design process, and it is shown in Fig 5 as steps 1*’*→3→6→7 in turn. The requirements for principle innovation are detailed in following part.

e) The process 4 in Fig 5 means that the requirements for structure innovation (*SR*) are mapped into structures (*S*). The structural design problem is modeled and then there obtains an innovative product design scheme according to the optimized or varied solution. This process can be shown in Fig 5 as steps 1*’’*→4→7 in turn. The requirements for structure innovation are detailed in following part.

**Table 2:** RFPS Process of innovative design.

No.	Process	Content
1	Extension analysis of requirements	The Requirements *R* are classified into *FR*, *PR* and *SR* based on the extension analysis methods, and the result can be clearly identified as *FR*, *PR* and *SR*.
2	From Requirements to Functions	The requirements of functions *FR* are corresponded to functions *F*.
3	From Requirements to Principles	The requirements of principles *PR* are corresponded to principles *P*.
4	From Requirements to Structures	The requirements of structures *SR* are corresponded to structures *S*.
5	Principle solutions of Function problems	The functions *F* are derived from *FR*, and it is to obtain a principle solution according to *FR*.
6	Structure solutions of Principle problems	The principles *P* are derived from *PR* and functions *F*, and it is to obtain a structure solution according to *PR*.
7	Design Scheme of Structures	The structures *S* are derived from *SR*, the product and the principle, and it is to obtain an innovative or optimized structure scheme according to *SR*.

### Function innovation process

**Fig 6:**
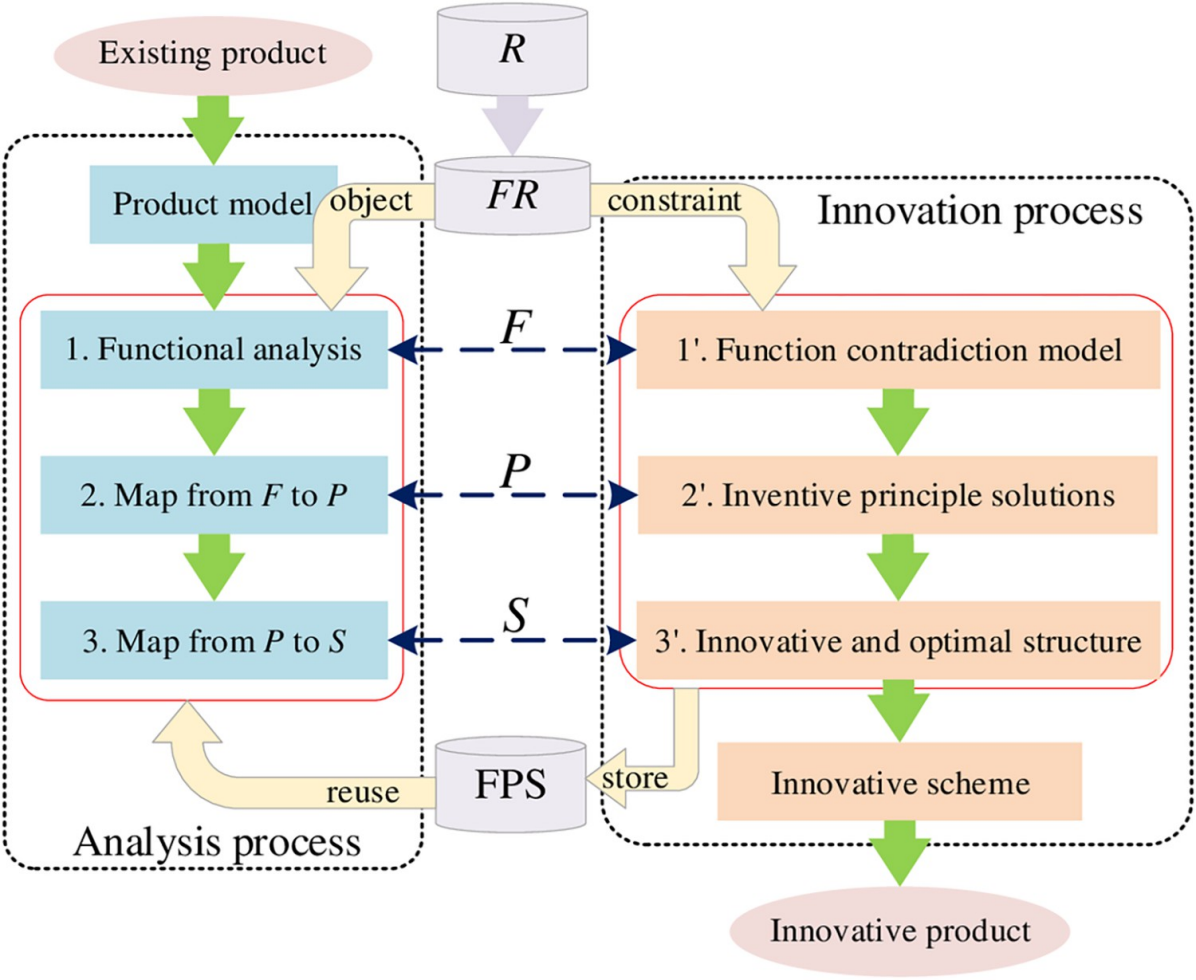
Function innovation process.

The design process for function innovation is shown in Fig 6, and there are two aspects–the analysis process based on RFPS design knowledge and the innovation process based on function innovation method.

The RFPS design knowledge of the initial product is generally stored in the early database and can be reused directly, and new RFPS design knowledge of the innovative product can be stored in database after decision of innovation process. The design process takes the model of the existing product and the innovation requirements *R*_*F*_ as inputs. The existing product model expresses the initial platform of the product. Generally, the RFPS design knowledge of the existing product is reused from databases by referring to the product model and the analysis process of mapping functions, originated from the requirements *FR*, to principles and then to structures. Function innovation ultimately focus on product structure. The specific steps of innovation process are as follows:

Step 1: Model the function innovation design as a technical contradiction or physical contradiction based on TRIZ;

Step 2: Obtain some inventive principle solution(s) by referring to the contradiction matrix with the inventive principles; and

Step 3: Determine the innovative structure entity and optimized parameters.

This innovation process can be formalized as:

$$FR\to \{F\overset{R}{↔}F^{\prime }\}\to \{P(F)\overset{F}{↔}P^{\prime }(F^{\prime })\}\to \{S(P)\overset{P}{↔}S^{\prime }(P^{\prime })\}\to D$$ (8)

### Principle innovation process

**Fig 7:**
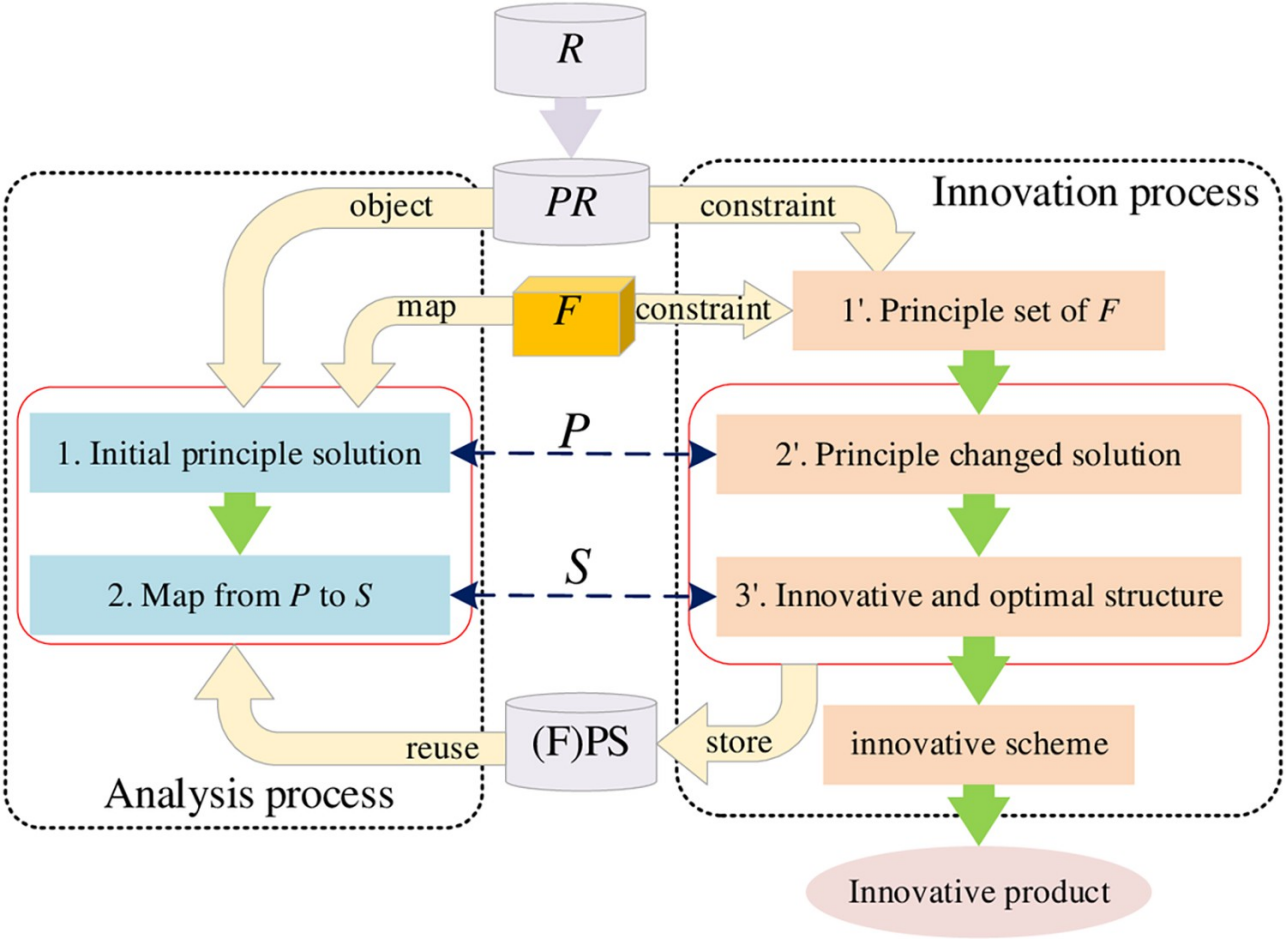
Principle innovation process.

The principle innovation of a product refers to that it does not change a function goal rather than changing the function principle. Therefore, it is necessary to determine the unchanged function goal before this kind of innovation process. The design process of *PR* is shown in Fig 7.

For analysis process of principle innovation, on the one hand, the initial principle solution stems from the requirement for principle innovation, and it reflects implementation of a certain function and is expressed by a specific structure, which means that the mapping relations constitute the RFPS design knowledge. RFPS design knowledge is stored in the database and can be reused directly. On the other hand, new RFPS design knowledge can be made with changed principle solution and structure variation to achieve the function goal, and the specific steps of innovation process are as follows:

Step 1′: Establish a set of principles according to the initial design principle solution under the function *F*;

Step 2′: Obtain a principle changed solution according to the principle requirements and the function constraints; and

Step 3′: Do variation and optimization of the structure, which mapped with the principle P, based on the principle changed solution.

This innovation process can be formalized as:

$$PR\to \{P(F)\overset{R+F}{↔}P^{\prime }(F^{\prime })\}\to \{S(P)\overset{P}{↔}S^{\prime }(P^{\prime })\}\to D$$ (9)

### Structure innovation process

**Fig 8:**
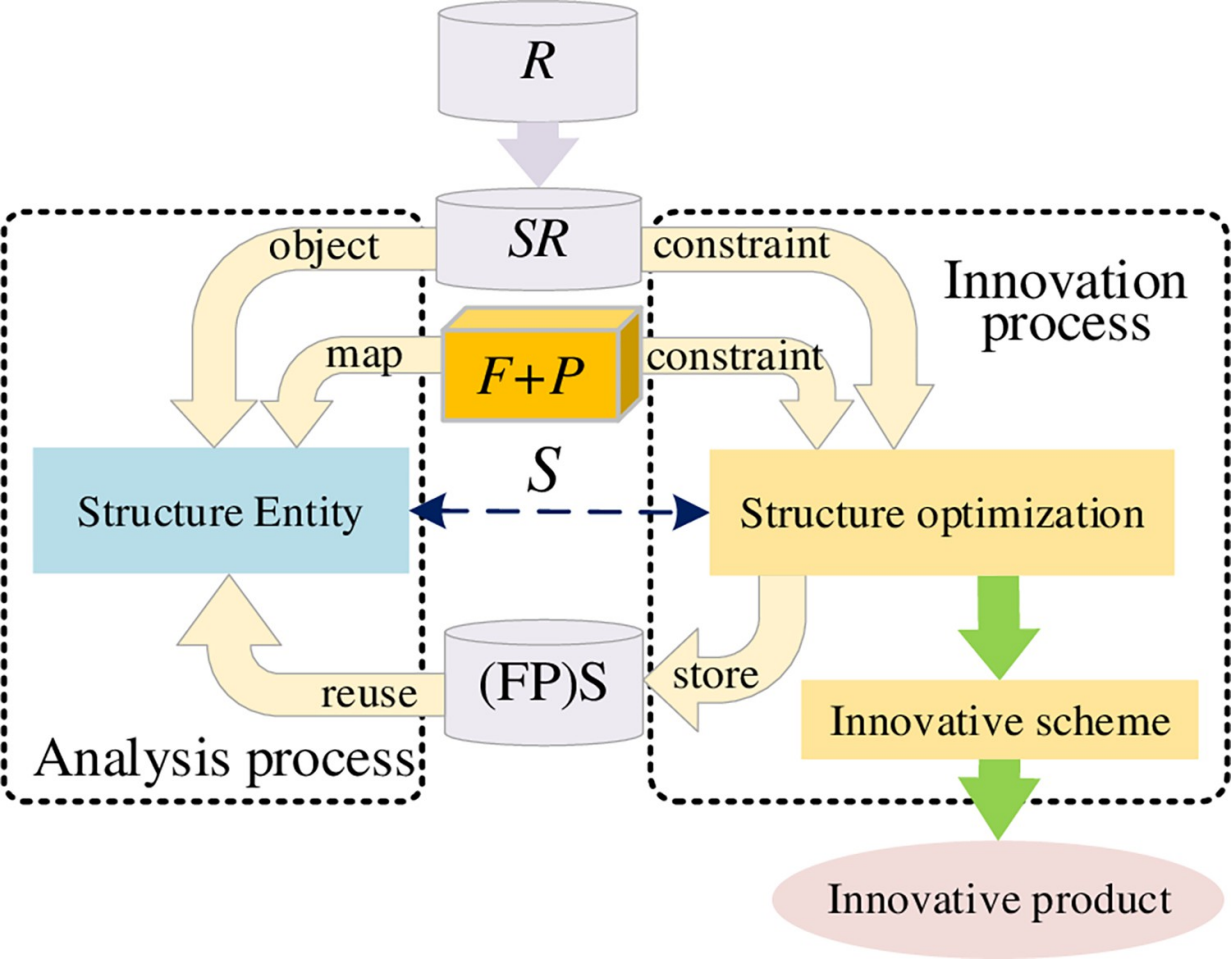
Structure innovation process.

Structure innovation is the easiest way to achieve product innovation and upgrade, and it is also direct and fast. The product structure innovation mainly focuses on the optimization of the material, size, shape, attributes, and characteristic parameters, etc. of the structure with certain constraints on the basis of the principles and functions determined. The design process of *SR* is shown in Fig 8.

The optimization of structure should not change functions or principle, but they should be used as constraints, which constitutes new RFPS design knowledge. RFPS design knowledge is stored in the database and can be reused directly. The specific steps of innovation process are as follows:

Step 1: Identify design variables *X* = {*x*_1_, *x*_2_, …, *x*_*n*_} of the structure entity;

Step 2: Determine objective function *f*(*X*) and constraints *g*(*X*) with the constant function and principle; and

Step 3: Choose an appropriate optimization method to get proper design parameters, such as genetic algorithm.

This innovation process can be formalized as:

$$SR\to \{S(F,P)\overset{R+F+P}{↔}S^{\prime }(F,P)\}\to D$$ (10)

## Case study

**Fig 9:**
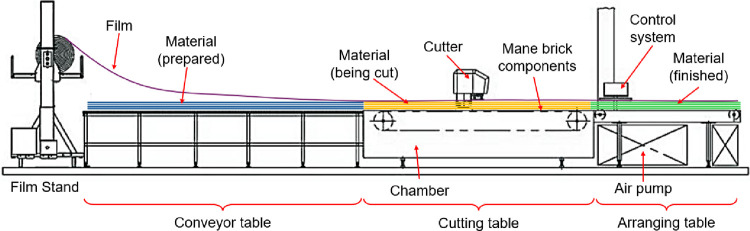
Operation schematic diagram of cutting table.

Take a CNC cutting table as an example to illustrate the innovative design process. The CNC cutting table is a complex product that integrates functions such as cutting, feeding, pressing, etc., shown as Fig 9. There are main working steps of the equipment as Table 3.

**Table 3:** Working steps of CNC cutting table.

Step	Process	Main Contents
1	Material preparation	The material, such as cloth or leather, has been stacked on a conveyor table to form a patch with a height of about 100 mm.
2	Feeding	The feeding system is activated to transport the material to the vicinity of the removal port, which is called the cutting window, and the feeding system is halted once one end of the material reaches near the outlet of the window.
3	Adsorption	the material is laminated by a film so that it is situated in a relatively closed environment, and then the air pump runs to pump out the air in the chamber under the film, so the different pressure causes between inside and outside that the chamber becomes a low-pressure environment, and the material is pressed on the cutting table by the atmosphere.
4	Cutting	The cutter system runs, and the material is cut to the desired shape along the path predefined according to the layout system (PC)
5	Material Removal	The cutter system is paused when the cutting finished, and reduce the pumping rate so that the air pressure in the chamber increases slightly, and the material adsorbed on the cutting table is easy to move. The feeding system runs to deliver the finished material to the arranging table, and the left prepared material to be cut is sent to the cutting table.

Steps 1 through 5 are repeated for the subsequent materials. Once all materials have been cut, the system is powered down, concluding the operation.

Evidently, the presence of idle periods during the material removal step adversely affects work efficiency. To bolster productivity, a strategic top-level design requirement has been proposed: the simultaneous execution from material feeding to cutting. This approach, which is called feeding cut, is intended to optimize the transition from material intake to the cutting process. Empirical evidence suggests that implementing this requirement could enhance production efficiency by a notable margin of 5% to 10%.

**Fig 10:**
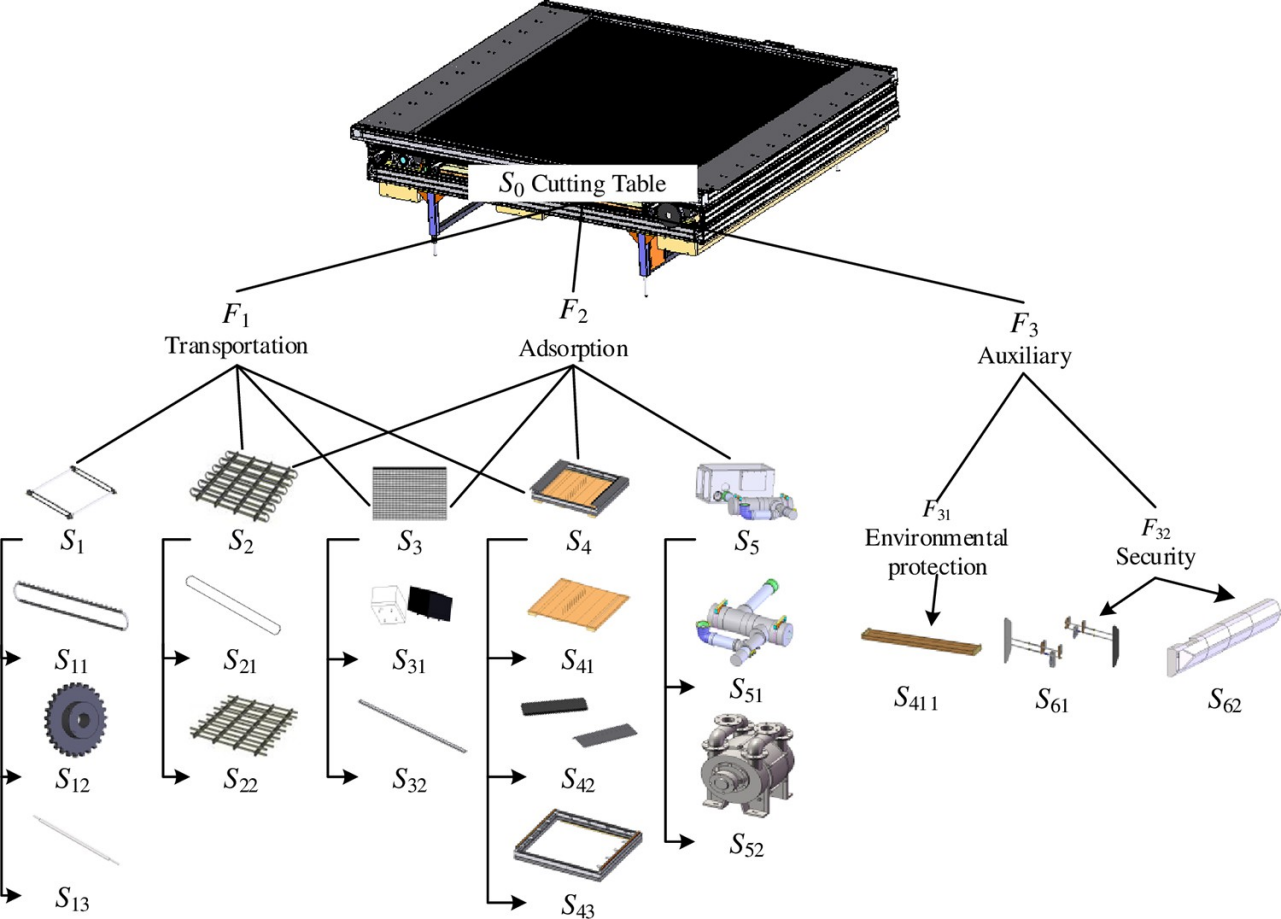
Cutting table and its structure decomposition. *S*_1_-Chain components; *S*_11_-Chain; *S*_12_-Sprocket; *S*_13_-Connecting rod; *S*_2_-Mane holder; *S*_21_-Rail; *S*_22_- Rail support; *S*_3_-Mane brick components; *S*_31_-Mane brick; *S*_32_-Brick Plate; *S*_4_-Chamber; *S*_41_-Chamber floor; *S*_411_-Waste box; *S*_42_-Inlet and outlet; *S*_43_-Chamber frame; *S*_5_-Pump components; *S*_51_-Pipeline; *S*_52_-Air pump; *S*_61_-Safty barriers; *S*_62_-Shell.

The main innovative structure is the cutting table for the design requirement. The structural decomposition of the cutting table is shown as Fig 10.

As described above, the requirement for innovation can be represented as *R*, and this is a top-level requirement. This requirement is analyzed as Eq (11) with extension analysis.


$$\left\{ \begin{array}{l} R┤\{RF_{1},RF_{2}\}⇐\{RF_{1},RF_{2},RF_{1}⊕RF_{2}\}\\ RF_{1}⇐\{RP_{1},RP_{2},RP_{3}\}┤\left\{ \begin{array}{l} RP_{1}┤\{RP_{11},RP_{12},...\}\\ RP_{1}⇐RS_{1}=\left\{ \begin{array}{l} RS_{1}⇐(RS_{11}\wedge RS_{12}\wedge RS_{13})\\ RS_{1}//\{RS_{11},RS_{12},RS_{13}\} \end{array}\right.\\ RP_{2}┤\{RP_{21},RP_{22}\}\\ RP_{2}⇐\{\left(RS_{2},RS_{3})\wedge (RS_{3},RS_{4}\right)\}┤\left\{ \begin{array}{l} RS_{2}=\left\{ \begin{array}{l} RS_{2}⇐(RS_{21}\wedge RS_{22})\\ RS_{2}//(RS_{21},RS_{22}) \end{array}\right.\\ RS_{3}=\left\{ \begin{array}{l} RS_{3}⇐(RS_{31}\wedge RS_{32})\\ RS_{3}//(RS_{31},RS_{32}) \end{array}\right.\\ RS_{4}=\left\{ \begin{array}{l} RS_{4}⇐(RS_{41}\wedge RS_{42}\wedge RS_{43})\\ RS_{4}//(RS_{41},RS_{42},RS_{43}) \end{array}\right. \end{array}\right.\\ RP_{3}⇐RS_{2}=\left\{ \begin{array}{l} RS_{2}⇐(RS_{21}\wedge RS_{22})\\ RS_{2}//(RS_{21},RS_{22}) \end{array}\right. \end{array}\right.\\ RF_{2}⇐\{RP_{4},RP_{5}\}┤\left\{ \begin{array}{l} RP_{4}⇐\{RS_{3},RS_{4}\}=\left\{ \begin{array}{l} RS_{3}=\left\{ \begin{array}{l} RS_{3}⇐(RS_{31}\wedge RS_{32})\\ RS_{3}//(RS_{31},RS_{32}) \end{array}\right.\\ RS_{4}=\left\{ \begin{array}{l} RS_{4}⇐(RS_{41}\wedge RS_{42}\wedge RS_{43})\\ RS_{4}//(RS_{41},RS_{42},RS_{43}) \end{array}\right. \end{array}\right.\\ RP_{5}⇐RS_{5}=\left\{ \begin{array}{l} RS_{5}⇐(RS_{51}\wedge RS_{52})\\ RS_{5}//(RS_{51},RS_{52}) \end{array}\right. \end{array}\right. \end{array}\right.$$ (11)

In Eq (11), the letters are requirement representations with basic elements, and they are as follows (where the characteristics and values of objects are omitted here):

a) the basic elements of functions requirement: *RF*_1_ = (Transport, …), *RF*_2_ = (Pressing, …);

b) the basic elements of principles requirement: *RP*_1_ = (Drive mode, ⋯), *RP*_2_ = (Friction source, …), *RP*_3_ = (Support mode,…), *RP*_4_ = (Seal mode,…), *RP*_5_ = (Pump mode,…), *RP*_11_ = (Chain drive mode,…), *RP*_12_ = (Belt drive mode,…), *RP*_21_ = (Pressure condition,…), *RP*_22_ = (Surface condition,…); and

c) the basic elements of structures requirement: *RS*_1_ = (Chain components,…), *RS*_2_ = (Mane holder,…), *RS*_3_ = (Mane brick components,…), *RS*_4_ = (Chamber,…), *RS*_5_ = (Pump components,…), *RS*_11_ = (Chain,…), *RS*_12_ = (Sprocket,…), *RS*_13_ = (Connecting rod,…), *RS*_21_ = (Rail,…), *RS*_22_ = (Support,…), *RS*_31_ = (Mane brick,…), *RS*_32_ = (Brick Plate,…), *RS*_41_ = (Chamber floor,…), *RS*_42_ = (Inlet and outlet,…), *RS*_43_ = (Chamber frame,…), *RS*_51_ = (Pipeline,…), *RS*_52_ = (Air pump,…).

In the case of non-feeding cut, the adsorption system is turned off. The chain (*S*_1_) drives the mane brick components (*S*_3_) to run on the mane holder (*S*_2_). At this time, the resistance is only related to the gravity of the mane brick (*S*_31_) and the friction coefficient between the rail support (*S*_22_) and the guide rail (*S*_21_), there is only static friction.

$$\left\{ \begin{array}{l} f_{1}=\mu (G_{F}+G_{M}+G_{S})\\ G_{F}=m_{F}glhn_{F}=250\times 10\times 2\times 2\times 80=800N\\ G_{M}=m_{M}gn_{r}n_{c}=0.6\times 10\times 22\times 22=2900N\\ G_{S}=m_{S}gn_{S}=5.2\times 10\times 22=1144N \end{array}\right.$$ (12)

here, *μ* is the dynamic friction coefficient between *S*_21_ and *S*_22_. *G*_*F*_ is the gravity of the material, *m*_*F*_ is the mass of material per unit, *g* = 10 is the gravitational acceleration, *l* and *h* are length and height of the material, and the *n*_*F*_ is the layer number of the material; *G*_*M*_ is the gravity of the mane bricks, *m*_*F*_ is the mass of mane brick, *n*_*c*_ and *n*_*r*_ are numbers of row and column of *S*_3_; *G*_*S*_ is the gravity of the rail support, *m*_*S*_ is the mass of rail support, *n*_*S*_ is numbers of rail support.

The friction coefficient is different with and without lubrication, and the result is

$$\left\{ \begin{array}{l} f_{1}=\mu_{1}(G_{F}+G_{M}+G_{S})=87.192N\\ f_{1}=\mu_{2}(G_{F}+G_{M}+G_{S})=1937.6N\\ \mu_{1}=0.018,\mathrm{with}\;\mathrm{lubrication}\\ \mu_{2}=0.400,\mathrm{without}\;\mathrm{lubrication} \end{array}\right.$$ (13)

*S*_1_, *S*_2_ and *S*_3_ are integrated and connected. when transporting materials, they are hindered by the guide rails. Based on the analysis above, the design requirement can be met by improving the function *F*_1_. Therefore, this design problem constitutes a requirement for function innovation, *RF*_1_ = (Transport, …), and Its corresponding function is *F* = (moving, object, material), which can be solved with the TRIZ, as described in function innovation process. It is found that when the driving force was increasing for better transportation, it was that the system reliability would be greatly decreased via testing, especially for the life of chain, and this is a technical contradiction. Do a retrieval of contradiction matrix, principles of invention can be obtained, shown as Table 4.

**Table 4:** Contradiction matrix.

Improved Parameter	Worsening Parameter	Inventive Principles
32 Ease of operation	27 Reliability	17- another dimension
		27 Cheap Short-Living Objects
		8-Anti-Weight
		40-Composite Materials

**Fig 11:**
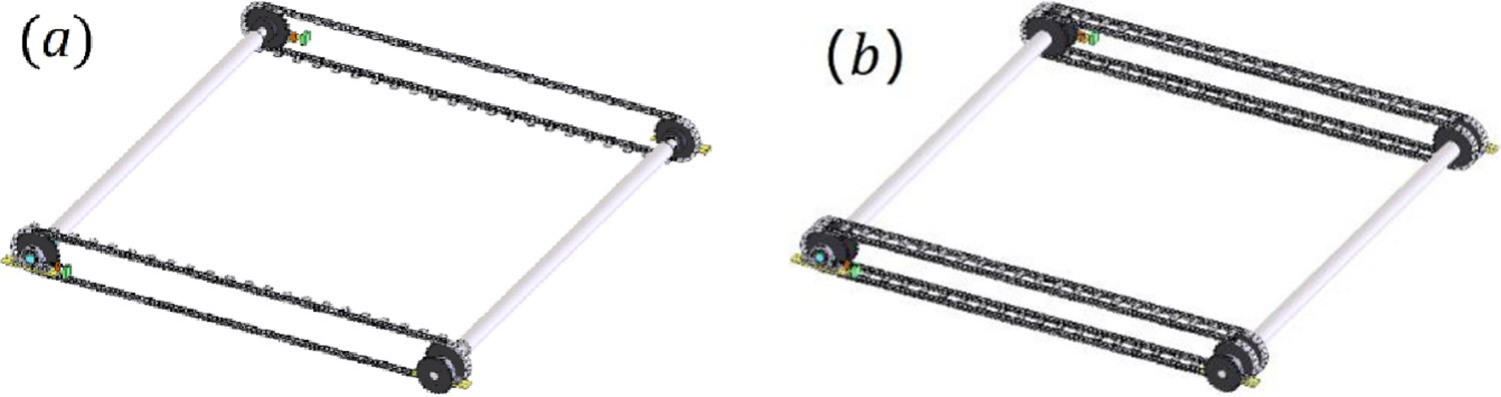
Scheme of drive chain. (a) before improvement; (b) after improvement.

Some structure schemes can be gotten based on these principles. According to a detail item in Principle 17 of the invention—make the objects multiple arrangements rather than single one, the multi-row chain can be used to disperse the tensile force of the chain and strengthen the overall performance. The design structure is shown in Fig 11. Another scheme can be obtained according to a detail item of principle 15 of the invention—make stationary objects movable. The rail structure, which is made of rigid resin material currently, can be converted into flexible materials and moves synchronously with the bristle bricks.

**Fig 12:**
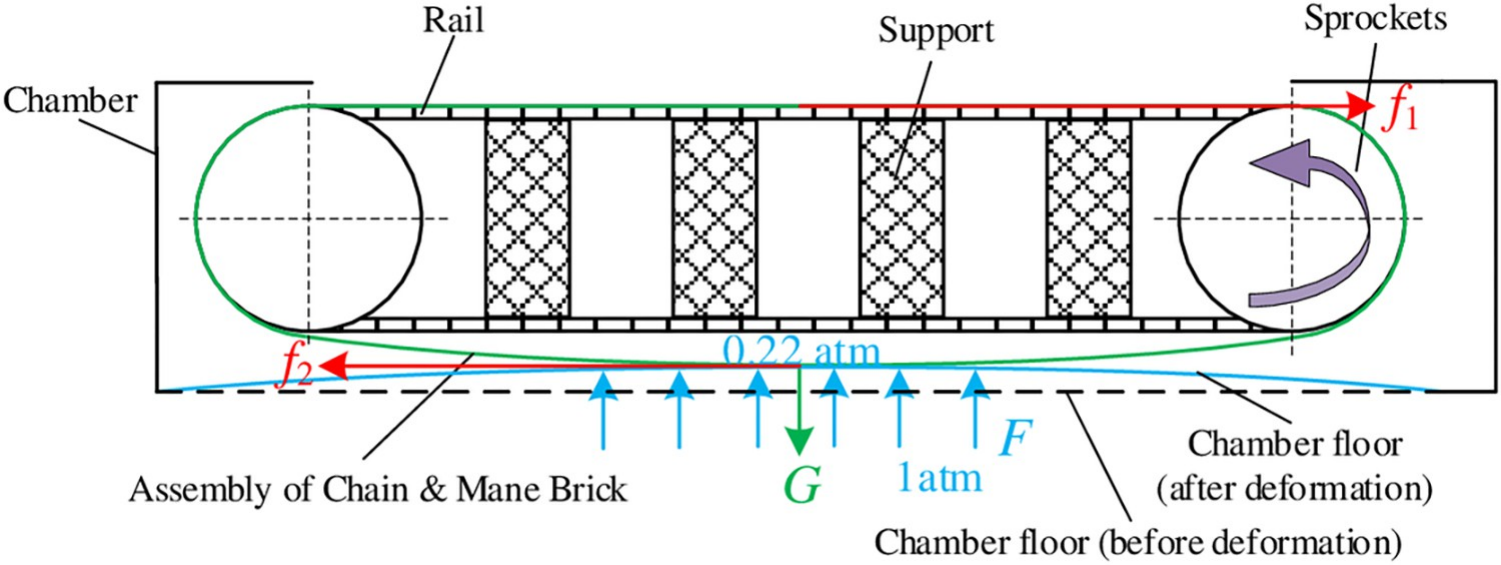
Two main friction of the cutting table during transportation.

In addition, the design requirement can be met by improving the principle *RP*_2_ in another way. Therefore, this design problem constitutes a requirement for principle innovation, which can be solved with the steps described in section 3.2. There are 2 main frictions of the cutting table during transportation to hinder sprocket movement. One is the sliding friction force *f*_1_ between the mane bricks and the support, and the other is the contact friction *f*_2_ between the falling bricks surface due to gravity and the deformed chamber floor due to the air pressure, as shown in Fig 12.

In the case of feeding cut, the adsorption system is turned on. From the perspective of product structure, the bricks are fixed with the chain, and it is obstructed by the rail and the chamber floor at the same time when the table running. Therefore, the structure pairs involved in the friction surfaces are (*S*_21_, *S*_32_) and (*S*_31_, *S*_41_), which generate friction forces *f*_1_ and *f*_2_ respectively. Considering the factors of air infiltration into the chamber after cutting, it can ensure that the material does not slip. the air adsorption force is *F* = 10867.106*N* when adsorbing, so the maximum friction force on the rail is

$$f_{1\max}=0.4\times (10867.106+800+2900+1144)=6284.443N$$ (14)

and the friction coefficient between the chamber floor and the mane brick is 0.6, so the resistance on the bottom is

$$f_{2\max}=0.6\times (10867.106+2900+1144)=8946.664N$$ (15)

so, the total resistance is

$$f=f_{1\max}+f_{2\max}=15231.107N$$ (16)

The friction of transportation with adsorption is 7.8 times greater than without adsorption.

**Fig 13:**
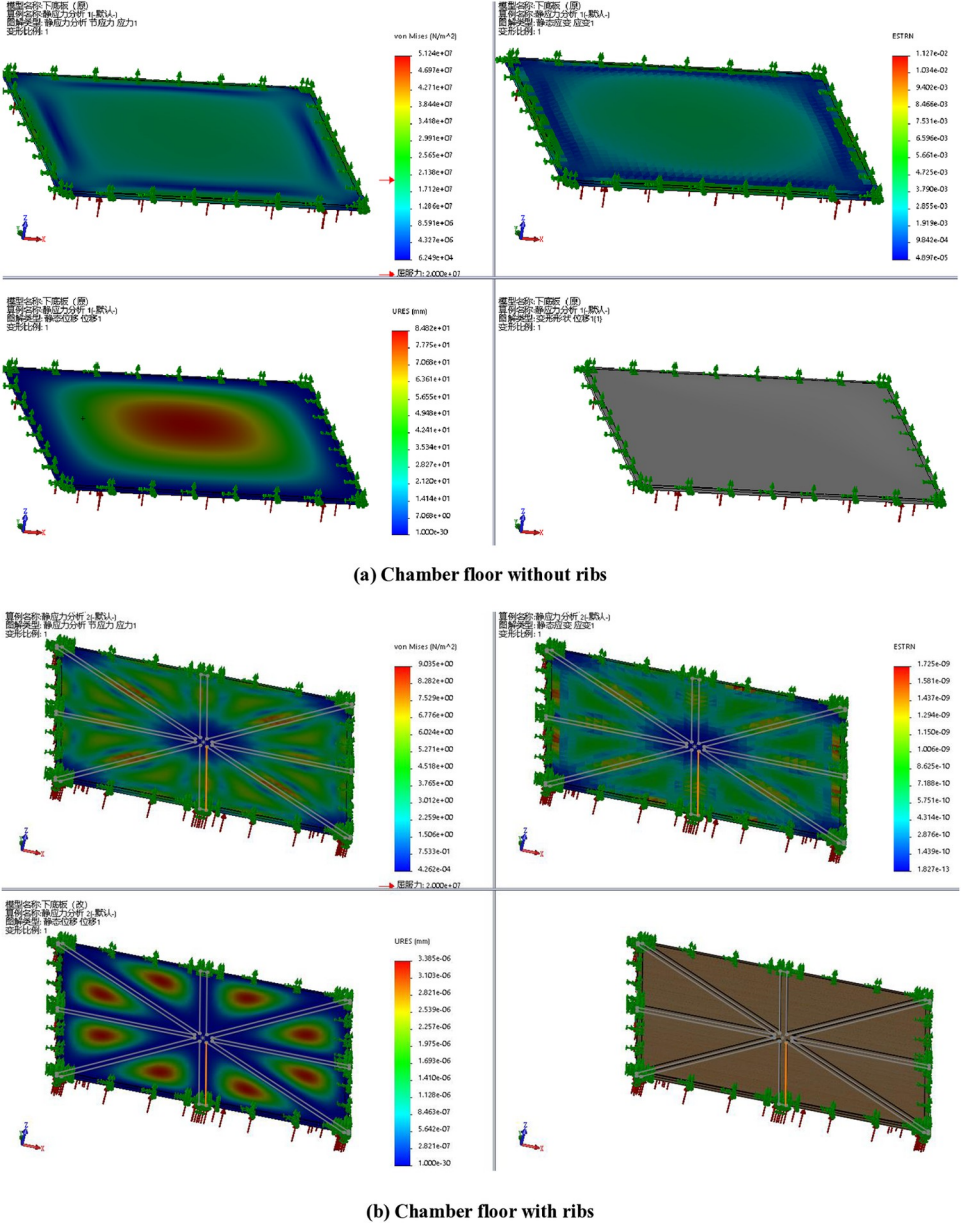
Simulation analysis of the chamber floor.

By searching the database, there are four basic principles to reduce friction by 1) reducing positive pressure; 2) reducing the roughness of the contact surfaces; 3) adding lubrication, air cushion or separation between the contact surfaces; and 4) Changing sliding to rolling. According to these principles, the friction force *f*_1_ can be improved by reducing the surface roughness, adding some lubricant or creating a gap between the brick plates (steel) and the rail (PC material). For the friction force *f*_2_, the third principle can be used to separate the bricks and chamber floor or reduce the contact area by adding reinforcing ribs to the chamber floor to reduce deformation, and the simulation shows that the range of deformation is reduced by an order of one, shown in Fig 13.

These are all conceptual schemes of product structures, so that it should be more improved in a structure optimization perspective. This paper will not go into detail, neither does the requirements for structure innovation.

## Discussion

This paper provides a RFPS framework to support innovation design from the perspective of different object of design requirements. It’s not a product design framework from scratch based on certain determined requirements, but an innovation or upgrade based on existing products according to the different requirements from all the parties.

This design methodology has some similarities and differences with some existing design methodologies. For example, the RFPS model and the function-behavior-structure (FBS) model both use key elements to represent design knowledge, but the RFPS model emphasizes more on the hierarchy and flexibility of requirements, as well as the extension analysis methods. Another example is that the RFPS model and the axiomatic design (AD) method both implement the design process by mapping between different design domains, but the RFPS model focuses more on the mapping from high-level requirements to low-level requirements, while the AD method focuses more on the mapping from functional requirements to design parameters. By comparing and analyzing the existing design methodologies, it can be seen that the innovation and advantages of the RFPS model, as well as its potential shortcomings and improvement space.

The main feature of the proposed methodology is that different design processes are adopted according to the different requirement objects, and the different requirement objects correspond to the function, principle and structure innovations respectively. From the start of the requirements for innovation or upgrade, the extension analysis methods were used to map the requirements to the functions, principles and structures. This is similar to decomposition and classification of the requirements, that is, the complex top-level requirements are decomposed into simple bottom-level ones, so that the bottom-level requirements can be classified according to the objects of innovation intention.

The other feature of the methodology is shown in Fig 5. RFPS knowledge management platform is a necessary computer-aided technology to store the design knowledge from innovation process into database for further use, although there are some default elements of FPS for principle and structure innovation. The different innovation processes were used to make the product upgrade, as the detail processes shown in Figs 6–8, which means the function, principle and structure are regarded equal for product innovation. On the one hand, different steps are applied to get the design scheme for each kind of innovation process, so that it may increase complexity to the design process; on the other hand, these steps are not unique, which means that other innovative methods can be used to obtain the schemes, and it will significantly increase the probability for product innovation. Given the non-uniqueness of the steps involved in the innovation processes and the equal emphasis on function, principle, and structure, the RFPS platform stands to benefit significantly from the implementation of more efficient and innovative design methodologies, which are crucial for navigating the intricacies of product innovation across different levels.

Design knowledge representation is an important part for knowledge-based design [28]. As described in section 2, the basic element is used to represent the knowledge of design object and some symbols are introduced to represent the analysis processes. There still needs some ways to represent the innovation process, the conduction transformation provides a viable way to describe the dynamic knowledge for the design process.

Another important missing piece is evaluation system [29], although some innovative design schemes based on the proposed methodology was succeeded in designing a cutting table, as described in section 4, there should be an evaluation system to evaluate these design results according to the time consumption, cost, and other considerations.

## Conclusion and future work

The redesign and remanufacturing of the outdated product are important way of reducing waste of resources to avoid the environmental problems. In addition, the upgradable existing product is the most common innovation design object. In this paper, a systematic design methodology for product innovation and upgrade is proposed. The extension analysis methods are introduced to map the requirements to function, principle and structure requirements; and RFPS methodology supports designers to choose a suitable process to deal with the uncertain complex requirements. This paper mainly focused on the analysis and innovation operations, and the case study verified feasibility of the methodology. Due to the important role of knowledge representation in conceptual design, the proposed method, which refers to an Extenics-TRIZ integrated RFPS knowledge representation model that combines different requirement objects and the top-to-bottom requirement analysis, can not only illustrate the mapping relations between requirements and function, principle and structure, but also enhance the internal knowledge representation of each layer and the knowledge reuse.

In light of the limitations mentioned in the discussion, there are identified several avenues for future research to address these shortcomings and further advance our work:

a) Development of a Comprehensive RFPS Knowledge Management Platform. It is necessary to construct a systematic platform for RFPS knowledge management. This platform will serve to consolidate design knowledge across various product types and will feature a graphical interface that enhances the visualization and interaction with both design knowledge and product entity.

b) Integration of Advanced Design Methodologies. The future work will focus on integrating additional sophisticated design methodologies to enrich the innovation design process, such as the Taguchi Methods [30], which are recognized for their ability to bolster quality management within design processes. The incorporation of these methods is expected to elevate the caliber of our design outcomes.

c) Establishment of a evaluation system. The evaluation system encompasses all proposed schemes., and it will be anchored in the principles of innovation, economic viability, and environmental sustainability, providing a robust framework for assessing the multifaceted success of our design strategies.
